# Development of the Japanese version of the Council on Nutrition Appetite Questionnaire and its simplified versions, and evaluation of their reliability, validity, and reproducibility

**DOI:** 10.1016/j.je.2016.11.002

**Published:** 2017-02-03

**Authors:** Yuko Tokudome, Keiko Okumura, Yoshiko Kumagai, Hirohiko Hirano, Hunkyung Kim, Shiho Morishita, Yutaka Watanabe

**Affiliations:** a Graduate School of Nutritional Sciences, Nagoya University of Arts and Sciences, Aichi, Japan; b Department of Education and Research in Family and Community Medicine, Mie University Graduate School of Medicine, Mie, Japan; c School of Nutritional Sciences, Nagoya University of Arts and Sciences, Aichi, Japan; d Department of Dentistry and Oral Surgery, Tokyo Metropolitan Geriatric Hospital and Institute of Gerontology, Tokyo, Japan; e Research Team for Promoting Independence of the Elderly, Tokyo Metropolitan Institute of Gerontology, Tokyo, Japan; f Department of Oral Disease Research, National Center for Geriatrics and Gerontology, Aichi, Japan

**Keywords:** Appetite, Development of questionnaires, Japanese elderly, Reliability, Validity

## Abstract

**Background:**

Because few Japanese questionnaires assess the elderly's appetite, there is an urgent need to develop an appetite questionnaire with verified reliability, validity, and reproducibility.

**Methods:**

We translated and back-translated the Council on Nutrition Appetite Questionnaire (CNAQ), which has eight items, into Japanese (CNAQ-J), as well as the Simplified Nutritional Appetite Questionnaire (SNAQ-J), which includes four CNAQ-J-derived items. Using structural equation modeling, we examined the CNAQ-J structure based on data of 649 Japanese elderly people in 2013, including individuals having a certain degree of cognitive impairment, and we developed the SNAQ for the Japanese elderly (SNAQ-JE) according to an exploratory factor analysis. Confirmatory factor analyses on the appetite questionnaires were conducted to probe fitting to the model. We computed Cronbach's α coefficients and criterion-referenced/-related validity figures examining associations of the three appetite battery scores with body mass index (BMI) values and with nutrition-related questionnaire values. Test–retest reproducibility of appetite tools was scrutinized over an approximately 2-week interval.

**Results:**

An exploratory factor analysis demonstrated that the CNAQ-J was constructed of one factor (appetite), yielding the SNAQ-JE, which includes four questions derived from the CNAQ-J. The three appetite instruments showed almost equivalent fitting to the model and reproducibility. The CNAQ-J and SNAQ-JE demonstrated satisfactory reliability and significant criterion-referenced/-related validity values, including BMIs, but the SNAQ-J included a low factor-loading item, exhibited less satisfactory reliability and had a non-significant relationship to BMI.

**Conclusions:**

The CNAQ-J and SNAQ-JE may be applied to assess the appetite of Japanese elderly, including persons with some cognitive impairment.

## Highlights

• We translated the CNAQ and SNAQ into Japanese (CNAQ-J and SNAQ-J).
• The CNAQ-J was found to be constructed of one factor (appetite).
• We developed the SNAQ-JE according to an exploratory factor analysis.
• The CNAQ-J and SNAQ-JE showed acceptable reliability, reproducibility, and validity.
• The CNAQ-J and SNAQ-JE may be applied to assess Japanese elderly's appetite.

## Introduction

In Japan, the prevalence of under-/malnutrition or body mass index (BMI) ≤20 was reported to be 16.8% among the community-dwelling elderly ≥65 years of age according to the National Health and Nutrition Survey, 2013.^1^ The estimated prevalence in adults ≥85 years of age was reported to be 29.6%, and the figure among patients institutionalized in health-care facilities was 77.9%.^2^

One of the major factors leading to deterioration of a healthy life among the elderly seems to be under-/malnutrition, which is triggered by loss of appetite (LOA; i.e., anorexia) caused not only by age-related physiological factors, including degraded/impaired oral health, sense of smell and taste, digestive functions, and physical activity,^3^, ^4^, ^5^, ^6^, ^7^ but also by dysfunctions of clinical parameters, including mental/psychological disorders (such as dementia and depression), and effects/side effects of medicines.^8^, ^9^, ^10^, ^11^ Under-/malnutrition is also associated with socioeconomic factors, including living alone and sparse or loss of family relationships and social/community communication.^12^, ^13^

Furthermore, LOA itself inevitably worsens activities of daily living (ADL) and quality of life (QOL) because meals are basic to as well as prerequisite for enjoyment of life, especially for the elderly. To adequately evaluate appetite seems critical; however, Japanese appetite questionnaires used currently are comprised of one or two yes/no questions, without evidence of reliability, validity, or reproducibility.

In 2005, using the Delphi method, Wilson et al^14^ developed the Council on Nutrition Appetite Questionnaire (CNAQ), which includes eight items (eTable 1). Conducting studies on a long-term care group (mean [standard deviation {SD}] age 79.2 [9.0] years) and a community-dwelling group (mean [SD] age 53.5 [20.2] years, including an elderly group [age range >60–102 years] and a young and elderly group [age range >20–60 years]), they verified its reliability and concurrent validity using an external lengthy assessment tool (the Appetite, Hunger and Sensory Perception Questionnaire)^15^ as the reference standard. The CNAQ and the Simplified Nutritional Appetite Questionnaire (SNAQ), which retains items #1, #2, #4, and #6 from the CNAQ, are now used for young and elderly people (including patients) worldwide in countries including Australia, Malaysia, Germany, and Korea.^16^, ^17^, ^18^, ^19^

In the present study, we translated and back-translated the original CNAQ and SNAQ into Japanese (the CNAQ-J and SNAQ-J, respectively) according to a standardized procedure and developed the SNAQ for the Japanese elderly (SNAQ-JE) using a series of exploratory factor analyses, and we studied fitting to the structural equation modeling (SEM), reliability, criterion-referenced/-related validity, and reproducibility to verify whether those appetite batteries can be applied to the Japanese elderly.

## Methods

### The original CNAQ and SNAQ

For convenience, we refer to CNAQ items as follows in this article: #1, Appetite; #2, Feeling full; #3, Feeling hunger; #4, Food tastes; #5, Food tastes compared to when younger; #6, Meal frequency per day; #7, Feel sick or nauseated when eating; and #8, Usual mood. The subjects were requested to reply using 1–5 ordinal scales (Likert scales) of each question.

The CNAQ (and corresponding SNAQ) scores of eight items were tallied, and the total scores ranged 8–40 (4–20 for SNAQ). Scores ≤28 (≤14 for SNAQ) may predict “at risk,” while scores ≥29 (≥15 for SNAQ) “low risk at this stage” of 5–10% (*ibid*) body weight loss from their baseline weight over a 6-month period with approximately 80% (70%–90%) sensitivity and specificity.

### Development of the Japanese versions of the CNAQ-J and SNAQ-J

We obtained permission from the original article's authors,^14^ including the senior author (JEM), for development of the Japanese versions. Using a standardized translation and back-translation method, a Japanese version (CNAQ-J) was developed by two nutrition researchers, a medical doctor, a professor of English, and a Japanese staff member majoring in English. Translation and back-translation were reiterated until equivalent expressions in English were attained. A pilot study of 15 people (aged 20 s–90 s, including an elderly person attending a health-care facility) was conducted to examine whether the Japanese expressions adopted for the CNAQ-J were understandable or not. Younger people were also invited to participate in this study because the younger generation can play roles as proxy/surrogate interview-based responders. Minor revisions were added, and the questionnaire was reckoned as feasible and applicable for the main study. The Japanese versions (the CNAQ-J and SNAQ-J) were finalized (eTables 2 and 3).

### Study subjects

From July to December 2013, we recruited 816 subjects, including 175 community-dwelling elderly (CE group) attending health promotion classes for a secondary prevention study to improve mild frailties in O City, Aichi Prefecture; 328 receiving meal delivery (MD group) services in N City, Aichi Prefecture; 163 attending day-care (DC group) facilities in O City, Fukuoka Prefecture, and T City, Toyama Prefecture; and 150 staying at group homes (GH group) in Y City, Kanagawa Prefecture.

For a test–retest reproducibility analysis, 54 elderly people (10 CE and 44 DC persons) were invited independently from the main study.

### Data collection

We gathered data on subjects' basic characteristics (gender and age), their anthropometric measurements (height, weight, and BMI), the CNAQ-J, the Clinical Dementia Rating (CDR)^20^, ^21^ (from the MD, DC, and GH groups), the Mini Nutritional Assessment-Short Form (MNA-SF)^2^, ^22^ (from the CE, DC, and GH groups), and the Constipation Assessment Scale-Japanese Version (CAS-J)^23^, ^24^ (from the CE and GH groups).

All data, except for anthropometric measurements, were collected using relevant appetite and nutrition-related questionnaires. We obtained self-administered replies from the CE group, self- and/or interview-administered replies by proxy caregivers/family members of the MD group, and self- and/or interview-administered replies by surrogate facility staff of DC and GH groups.

### Exploratory factor analyses of the appetite batteries, and development of the SNAQ-JE

We performed exploratory factor analyses of the CNAQ-J and SNAQ-J under the SEM using the maximum likelihood method, and developed the SNAQ-JE, taking into account the distribution of the CNAQ-J item scores, deleting any item having a smaller factor loading, reducing Cronbach's α coefficient, or deploying a higher brain function.

### Examination of fitting to the model

We conducted confirmatory factor analyses to scrutinize the goodness of fit index (GFI), adjusted GFI (AGFI), and a root mean square error of approximation (RMSEA) for the three appetite tools.

### Appraisal of reliability/internal consistency

We examined reliability/internal consistency of the CNAQ-J, SNAQ-J, and SNAQ-JE.

### Criterion-referenced/-related validity

Using the previously mentioned cutoff values of the CNAQ-J and SNAQ-J, along with cutoffs of ≤14 or ≥15 for the SNAQ-JE score (based on an average value 14.4), we categorized people into a lower-score group and a higher-score group. BMI values were contrasted between the two score groups in a cross-sectional manner, and correlations were probed between appetite questionnaire scores and nutrition-related questionnaire values.

### Test–retest reproducibility

We studied test–retest reproducibility of appetite questionnaire scores setting an approximately two-week interval.

### Statistical analyses

To compare baseline characteristics with the CNAQ-J item scores by subject group, we conducted *t*-tests for two-group comparisons, analysis of variance (ANOVA) with post hoc Bonferroni adjustment for multiple-group comparisons, and *χ*^2^ tests for comparisons of proportions.

Exploratory factor analyses of the appetite batteries were performed adopting the maximum likelihood method, and the SNAQ-JE was developed from these analyses.

Confirmatory factor analyses were then conducted to probe fitting to the model (including GFI, AGFI, and RMSEA) for the CNAQ-J, SNAQ-J, and SNAQ-JE.

Reliability/internal consistency was appraised calculating Cronbach's α coefficient.

For a criterion-referenced/-related validity study, the BMI difference between a higher-score group vs a lower-score group was examined by *t*-test. We also explored correlations of appetite questionnaire scores with nutrition-related instrument indices by age- and gender-adjusted Pearson correlation coefficients.

Test–retest reproducibility of appetite tool scores was verified adopting intraclass correlation coefficients (ICCs).

For statistical analyses, SPSS ver. 22 (IBM Corp., Armonk, NY, USA) and Amos were used, and P < 0.05 (two-tailed) was assumed as statistically significant.

### Ethical issues

The present study protocol was submitted to the Committee of Ethics and Conflict of Interest at the National Center for Geriatrics and Gerontology (Number of receipt #648) and approved. All participants/proxies/surrogates were fully informed about the study and gave written consent.

## Results

### Study subjects

After excluding 167 individuals (7 CE, 127 MD, 19 DC, and 14 GH group participants) having incomplete information from the 816 subjects recruited, we analyzed the data provided by 649 subjects (168 CE, 201 MD, 144 DC, and 136 GH group participants).

There were 230 men (35.4%) and 419 women (64.6%) (Table 1). The average age among men (77.2 [SD, 8.4] years) was younger than among women (82.3 [SD, 7.7] years). Average heights were 162.3 (SD, 6.7) cm for men and 147.3 (SD, 6.9) cm for women. Body weights were 59.1 (SD, 9.4) kg for men and 47.2 (SD, 9.1) kg for women. BMIs were 22.4 (SD, 3.1) kg/m^2^ for men and 21.8 (SD, 3.9) kg/m^2^ for women.

**Table 1:** Demographic and anthropometric characteristics, and CDR scores by gender and study group.

	All (n = 649)	Men (n = 230)	Women (n = 419)	P^a^	CE^b^ (n = 168)		MD^c^ (n = 201)		DC^d^ (n = 144)		GH^e^ (n = 136)		P^f^
	Mean (SD)	Mean (SD)	Mean (SD)		Mean (SD)		Mean (SD)		Mean (SD)		Mean (SD)		
Age (yrs)	80.4 (8.4)	77.2 (8.4)	82.3 (7.7)	<0.001	73.5 (5.9)	u,v,w^g^	81.2 (8.1)	u,y,z	83.6 (7.5)	v,y	84.4 (7.1)	w,z	<0.001
Height (cm)	152.6 (9.9)	162.3 (6.7)	147.3 (6.9)	<0.001	156.7 (9.3)	u,v,w	153.9 (9.4)	u,y,z	150.5 (10.2)	v,y	147.6 (8.6)	w,z	<0.001
Weight (kg)	51.5 (10.8)	59.1 (9.4)	47.2 (9.1)	<0.001	56.9 (10.5)	u,v,w	50.4 (10.4)	u,x	50.7 (10.7)	v,y	47.2 (9.3)	w,x,y	<0.001
BMI (kg/m^2^)	22.0 (3.7)	22.4 (3.1)	21.8 (3.9)	<0.001	23.1 (3.4)	u,v	21.2 (3.3)	u,w	22.3 (4.0)	w	21.7 (3.8)	v	<0.001
CDR score	MD + DC + GH groups				CE (n = 168)		MD (n = 201)		DC (n = 144)		GH (n = 136)		
	All (n = 481)	Men (n = 145)	Women (n = 336)										
0	22.7 (%)	27.8	20.5	0.087^h^	NA		0.3		34.1		0.0		<0.001^h^
0.5	19.6	21.6	18.8		NA		32.0		0.0		11.3		
1	34.4	27.8	37.3		NA		20.7		57.1		42.7		
2	15.3	13.6	16.0		NA		9.3		6.3		34.7		
3	8.0	9.1	7.5		NA		8.7		2.4		11.3		

CDR, Clinical Dementia Rating; CE, community-dwelling elderly; DC, day-care facilities; GH, group homes; MD, meal delivery; NA, not applicable; SD, standard deviation.

a Comparisons between sexes using t-test.

b Community-dwelling elderly attending health promotion classes.

c Elderly receiving meal delivery services.

d Elderly attending day-care facilities.

e Elderly staying at group homes.

f Comparisons across four study groups using ANOVA.

g Statistically significant across the same letters (u, v, w, x, y, and z) by ANOVA with post hoc Bonferroni adjustment.

h χ^2^ test.

The CDR study, in which the CE group was excluded, showed that the percentage of participants with no problem (score 0) was 22.7%, with dementia suspected (score 0.5) was 19.6%, and with overt dementia (score 1–3) was 57.7% (34.4% light, 15.3% moderate, and 8.0% severe dementia).

### The CNAQ-J scores

The average CNAQ-J score of all subjects was 29.3 (SD, 3.4) (Table 2). The figure of the GH group was highest 30.9 (SD, 3.3), followed by the DC group at 29.8 (SD, 2.6), the CE group at 28.9 (SD, 2.8), and the MD group at 28.2 (SD, 3.8).

**Table 2:** CNAQ-J scores by question item, gender, and study group.

Question item		All (n = 649)	Men (n = 230)	Women (n = 419)	P^a^	CE^b^ (n = 168)		MD^c^ (n = 201)		DC^d^ (n = 144)		GH^e^ (n = 136)		P^f^
		Mean (SD)	Mean (SD)	Mean (SD)		Mean (SD)		Mean (SD)		Mean (SD)		Mean (SD)		
#1	Appetite	3.5 (0.8)	3.5 (0.8)	3.5 (0.8)	0.746	3.4 (0.7)	u,v^g^	3.4 (0.8)	w,x	3.7 (0.7)	u,w	3.8 (0.7)	v,x	<0.001
#2	Feeling full	3.8 (0.6)	3.8 (0.7)	3.7 (0.6)	0.748	3.7 (0.6)	u	3.7 (0.7)	v,w	3.8 (0.5)	v	3.9 (0.5)	u,w	<0.001
#3	Feeling hungry	2.8 (1.0)	2.9 (1.0)	2.8 (1.0)	0.167	2.8 (0.9)		2.9 (1.0)		2.7 (1.2)		2.9 (0.8)		0.115
#4	Food tastes	3.6 (0.7)	3.6 (0.7)	3.7 (0.7)	0.088	3.5 (0.6)	u,v,w	3.4 (0.7)	u,x,y	3.9 (0.7)	v,x	3.8 (0.6)	w,y	<0.001
#5	Food tastes compared to when younger	3.1 (0.6)	3.0 (0.6)	3.1 (0.6)	0.819	3.1 (0.6)		3.0 (0.6)		3.0 (0.5)	u	3.2 (0.7)	u	<0.01
#6	Meal frequency a day	4.0 (0.5)	4.0 (0.5)	4.0 (0.4)	0.246	4.0 (0.4)	u	4.0 (0.6)	v	4.2 (0.5)	u,v,w	4.0 (0.1)	w	<0.001
#7	Feeling sick or nauseated when eating	4.6 (0.6)	4.5 (0.6)	4.7 (0.6)	0.018	4.6 (0.5)	u,v	4.4 (0.8)	u,w,x	4.8 (0.4)	v,w	4.7 (0.7)	x	<0.001
#8	Usual mood	3.7 (0.7)	3.7 (0.6)	3.6 (0.7)	0.238	3.7 (0.6)		3.5 (0.7)	u	3.7 (0.7)	u	3.6 (0.8)		<0.05
	Total	29.3 (3.4)	29.1 (3.4)	29.4 (3.3)	0.339	28.9 (2.8)	u	28.2 (3.8)	v,w	29.8 (2.6)	v,x	30.9 (3.3)	u,w,x	<0.001

CE, community-dwelling elderly; DC, day-care facilities; GH, group homes; MD, meal delivery; SD, standard deviation.

a Comparisons between sexes using t-test.

b Community-dwelling elderly attending health promotion classes.

c Elderly receiving meal delivery services.

d Elderly attending day-care facilities.

e Elderly staying at group homes.

f Comparisons across four study groups using ANOVA.

g Statistically significant across the same letters (u, v, w, x, y, and z) by ANOVA with post hoc Bonferroni adjustment.

### Examination of the CNAQ-J structure and development of the SNAQ-JE

An exploratory factor analysis demonstrated that Eigenvalues attenuated as follows: 2.921, 1.006, 0.862, and so forth. Thus, a one-factor (interpreted as appetite) solution appeared reasonable.

First, item #7 exhibited a ceiling effect and was excluded. An exploratory factor analysis of the remaining 7 items deleted item #6 because its factor loading was 0.248 (<0.4). The next analysis showed that all items had factor loading >0.4, but item #3 was omitted since it was a Cronbach's α coefficient reducer. Item #5 (Food tastes compared to when younger), which required the use of memory and judgment of the past, was considered to be inadequate for the elderly with higher brain dysfunction. The SNAQ-JE ultimately consisted of 4 items (#1, #2, #4, and #8) (eTable 4).

### Comparison of the results of exploratory factor analyses

The factor loadings of item #6 were 0.257 and 0.279 for the CNAQ-J and SNAQ-J, respectively, and the factor loading values for the SNAQ-JE were all >0.5 (Table 3). The explained variances for respective factor 1 were 28.22, 33.66, and 37.70 for the CNAQ-J, SNAQ-J, and SNAQ-JE, in that order.

**Table 3:** Comparison of the results of exploratory factor analyses.

Question item^a^		CNAQ-J	SNAQ-J	SNAQ-JE
		Factor 1^b^	Factor 1	Factor 1
		Factor loading	Factor loading	Factor loading
#1	Appetite	0.675	0.760	0.715
#4	Food tastes	0.664	0.600	0.633
#2	Feeling full	0.619	0.576	0.587
#5	Food tastes compared to when younger	0.522		
#8	Usual mood	0.494		0.500
#7	Feel sick or nauseated when eating	0.476		
#3	Feeling hunger	0.410		
#6	Meal frequency a day	0.257	0.279	
Sum of (factor loading values)^2^		2.258	1.346	1.507
Explained variance (%) for respective factor 1		28.22	33.66	37.70

CNAQ-J, Council on Nutrition Appetite Questionnaire; SNAQ-J, Simplified Nutritional Appetite Questionnaire; SNAQ-JE, Simplified Nutritional Appetite Questionnaire for Japanese Elderly.

a Listed according to the order of factor loading values of the CNAQ-J.

b Factor extraction method: Maximum likelihood method.

### Fitting to the model

The GFI and AGFI values for the CNAQ-J, SNAQ-J, and SNAQ-JE were all >0.9, exhibiting a good fit to the model using confirmatory factor analyses (Table 4). The RMSEAs of the CNAQ-J, SNAQ-J, and SNAQ-JE were 0.063, <0.001, and 0.085, respectively.

**Table 4:** Comparison of values for fitting to the structural equation model.

	GFI	AGFI	RMSEA
CNAQ-J	0.973	0.951	0.063
SNAQ-J^a^	0.999	0.998	<0.001
SNAQ-JE^b^	0.991	0.955	0.085

AGFI, adjusted goodness of fit index; CNAQ-J, Council on Nutrition Appetite Questionnaire; GFI, goodness of fit index; RMSEA, root mean square error of approximation; SNAQ-J, Simplified Nutritional Appetite Questionnaire; SNAQ-JE, Simplified Nutritional Appetite Questionnaire for Japanese Elderly.

a Including items #1, #2, #4, and #6 of CNAQ-J.

b Including items #1, #2, #4, and #8 of CNAQ-J.

### Reliability/internal consistency

Cronbach's α coefficients for all subjects were 0.733, 0.640, and 0.700 for the CNAQ-J, SNAQ-J, and SNAQ-JE, respectively (Table 5). Cronbach's α coefficients by sex and study group for the SNAQ-JE were uniformly greater than those of the SNAQ-J.

**Table 5:** Cronbach's α coefficients by appetite questionnaire and study group.

Appetite questionnaire	All	Men	Women	CE^a^	MD^b^	DC^c^	GH^d^
	(n = 649)	(n = 230)	(n = 419)	(n = 168)	(n = 201)	(n = 144)	(n = 136)
CNAQ-J	0.733	0.731	0.735	0.668	0.810	0.507	0.771
SNAQ-J^e^	0.640	0.599	0.665	0.560	0.702	0.408	0.645
SNAQ-JE^f^	0.700	0.683	0.711	0.598	0.773	0.507	0.740

CE, community-dwelling elderly; DC, day-care facilities; GH, group homes; MD, meal delivery; SD, standard deviation.

a Community-dwelling elderly attending health promotion classes.

b Elderly receiving meal delivery services.

c Elderly attending day-care facilities.

d Elderly staying at group homes.

e Including items #1, #2, #4, and #6 of CNAQ-J.

f Including items #1, #2, #4, and #8 of CNAQ-J.

### Criterion-referenced/-related validity

The lower-score group according to the CNAQ-J and SNAQ-JE, but not the SNAQ-J, had significantly lower BMIs compared with a higher-score group (P < 0.01) (Table 6A, Table 6B). CNAQ-J scores were significantly correlated to the MNA-SF values (r = 0.124) and to the CAS-J values (r = −0.335). SNAQ-J scores were significantly associated with the CAS-J indices (r = −0.314), but not with the MNA-SF values. SNAQ-JE scores were significantly correlated with the MNA-SF values (r = 0.178) and the CAS-J figures (r = −0.357).

**Table 6A:** Criterion-referenced/-related validity analyses. Comparison of BMI values according to appetite questionnaire score.

	CNAQ-J			SNAQ-J^a^			SNAQ-JE^b^		
	n	Mean	SD	n	Mean	SD	n	Mean	SD
Lower-score group^c^	233	21.6	3.4	222	21.7	3.6	267	21.6	3.6
Higher-score group^d^	414	22.3	3.8	425	22.2	3.7	380	22.3	3.7
*p*^e^	0.009			0.063			0.008		

CNAQ-J, Council on Nutrition Appetite Questionnaire; SD, standard deviation; SNAQ-J, Simplified Nutritional Appetite Questionnaire; SNAQ-JE, Simplified Nutritional Appetite Questionnaire for Japanese Elderly.

a Including items #1, #2, #4, and #6 of CNAQ-J.

b Including items #1, #2, #4, and #8 of CNAQ-J.

c The score of CNAQ ≦ 28, SNAQ and SNAQ-JE ≦ 14.

d The score of CNAQ ≧ 29, SNAQ and SNAQ-JE ≧ 15.

e Using *t*-test.

**Table 6B:** Criterion-referenced/-related validity analyses. Pearson correlation coefficient^a^ of appetite questionnaire scores with nutrition-related questionnaire indices.

	Nutrition-related questionnaire			
	MNA-SF	*p*	CAS-J	*p*
	(n = 448)		(n = 304)	
CNAQ-J	0.124	0.030	－0.335	<0.001
SNAQ-J^b^	0.091	0.113	－0.314	<0.001
SNAQ-JE^c^	0.178	0.002	－0.357	<0.001

CAS-J, Constipation Assessment Scale-Japanese Version; CNAQ-J, Council on Nutrition Appetite Questionnaire; MNA-SF, Mini Nutritional Assessment-Short Form; SNAQ-J, Simplified Nutritional Appetite Questionnaire; SNAQ-JE, Simplified Nutritional Appetite Questionnaire for Japanese Elderly.

a Age- and gender-adjusted value.

b Including items #1, #2, #4, and #6 of CNAQ-J.

c Including items #1, #2, #4, and #8 of CNAQ-J.

### Test–retest reproducibility

For 54 elderly people independently recruited for a separate test–retest reproducibility sub-study (10 CE persons [aged 73.9 {SD, 4.2} years] and 44 DC persons [aged 80.4 {SD, 4.2}] years), the ICCs of test–retest reproducibility for the CNAQ-J, SNAQ-J, and SNAQ-JE were 0.787, 0.693, and 0.702, respectively (*P* < 0.001) (Table 7).

**Table 7:** Test–retest reproducibility of appetite questionnaire scores in community-dwelling elderly^a^ and elderly attending day-care facilities^b^ (n = 54).

Appetite questionnaire	Score at first self-administration/interview	Score at second self-administration/interview	ICC	*P*
	Mean (SD)	Mean (SD)		
CNAQ-J	29.5 (3.2)	28.6 （3.4）	0.787	<0.001
SNAQ-J^c^	15.2 (1.5)	14.8 (1.7)	0.693	<0.001
SNAQ-JE^d^	15.0 (2.0)	14.7 (2.0)	0.702	<0.001

ICC, intraclass correlation coefficient; SD, standard deviation.

a Community-dwelling elderly (n = 10, aged 73.9 (4.2)).

b Elderly attending *day-care* facilities (*n* = 44, aged 80.4 (4.2)).

c Including items #1, #2, #4, and #6 of CNAQ-J.

d Including items #1, #2, #4, and #8 of CNAQ-J.

## Discussion

We translated and back-translated the CNAQ into Japanese (the CNAQ-J), along with its simplified version (the SNAQ-J). An exploratory factor analysis demonstrated that the CNAQ-J was constructed of one factor (appetite). According to a step-by-step exploratory factor analysis, we developed the SNAQ-JE, which uses four questions derived from the CNAQ-J. The CNAQ-J, SNAQ-J, and SNAQ-JE showed similar fitting to the model and test–retest reproducibility. The CNAQ-J and SNAQ-JE demonstrated satisfactory reliability and significant criterion-referenced/-related validity values with BMIs, whereas the SNAQ-J manifested less satisfactory reliability, and non-significant criterion-referenced/-related validity with BMIs or MNA-SF values. Additionally, the SNAQ-J included item #6, which had a factor loading of only 0.279. Thus, the SNAQ-JE, instead of the SNAQ-J, may be better suited for use among Japanese elderly.

The SNAQ-JE reached a satisfactory level of Cronbach's α coefficient (0.700) in the present study, but the SNAQ-J did not (0.640). The Cronbach's α coefficient of the SNAQ-J in the present study was comparable to figures for a long-term care group in the original SNAQ study (0.51),^14^ geriatric patients in Malaysia (0.58), Brazilian participants in the Cardiopulmonary and Metabolic Rehabilitation Program (0.61), and community-dwelling Japanese elderly (0.55).^25^, ^26^, ^27^ The lower internal consistency values may be due to the fact that most replies to item #6 were aggregated to “three meals a day” (>80% of responses in the present study) with smaller variance, reflecting in part its low factor loading value (<0.4); indeed, the reliability figure improved when the item was excluded.^26^

LOA unduly drives down meal amount, causing weight loss and frailty in the elderly. Wilson et al^14^ reported that a CNAQ score ≤28 (the SNAQ score ≤14) may predict being “at risk” of a 5%–10% weight reduction with approximately 80% sensitivity and specificity. Due to the limited observation period and research scheme, we were unable to assess sensitivity and specificity of the SNAQ-JE. Instead, using the SNAQ-JE average value (mean) of 14.4, we observed significantly reduced BMIs among the lower SNAQ-JE score (≤14) group compared with those of the higher-score (≥15) group, but not for the SNAQ-J, in a cross-sectional analysis. Thus, the scores ≤14 and ≥ 15 could be used for the SNAQ-JE cutoff values.

As noted, the lower-score groups had significantly lower BMI values compared with respective higher-score groups on the CNAQ-J and SNAQ-JE, but not on the SNAQ-J. We observed that the three appetite battery scores were negatively associated with the CAS-J values, with statistical significance. The SNAQ-J and SNAQ-JE scores were significantly correlated with MNA-SF values (with age- and gender-adjusted Pearson correlation coefficients of 0.124 and 0.178, respectively), although both correlation coefficients were <0.3 observed in Japanese elderly people.^27^ The SNAQ-JE acceptability as a screening instrument should be further investigated to verify the test performance (sensitivity, specificity, and receiver-operating characteristic [ROC] curve analyses) in comparison with nutrition-related questionnaires, including the Malnutrition Universal Screening Tool (MUST) and the Seniors in the Community: Risk Evaluation for Eating and Nutrition (SCREEN)^14^, ^28^, ^29^ in addition to the MNA-SF.

Wilson et al^14^ developed the SNAQ by deleting “reliability reducers” (supported by a principal components analysis) from the CNAQ, but the authors included item #6, which had a skewed distribution and a factor loading <0.4 in the present study. We developed the SNAQ-JE using a series of exploratory factor analyses, deleting the items showing a ceiling effect, having a smaller factor loading, being an internal consistency reducer, and requiring the use of a higher brain function. Ultimately, fitting to the model, associations with BMIs and CAS-J scores, and reproducibility values were almost equivalent between the SNAQ-J and SNAQ-JE, but the SNAQ-JE reliability and criterion-referenced/-related validity compared to MNA-SF values were uniformly and consistently (significantly/non-significantly) more favorable than those of the SNAQ-J, which included the low-factor-loading item #6. Thus, the evidence-based SNAQ-JE (including items #1, #2, #4, and #8) may be adopted to assess the appetite of other ethnic elderly people worldwide instead of the original SNAQ, although the instrument was primarily developed for the Japanese elderly.

In view of public health nutrition, it appears critical to manage the appetite of elderly people to detect LOA and to prevent LOA-related disorders/syndromes in the early phase using pertinent appetite questionnaires. Wilson et al^14^ conducted surveys on a wide range of people (aged >20–102 years) but excluded persons having moderate-to-severe dementia (Mini-Mental State Examination [MMSE] score <18), because their instruments were self-administered by the subjects. However, biases may still exist when obtaining information from the elderly with impaired cognitive function and obtaining replies with proxy/surrogate interview-based assistance. Question items should be *a priori* confined to basic physiologic perception related to an appetite, excluding items requiring deployment of a higher brain function. Meanwhile, reports have demonstrated that the proxy/surrogate respondent's information on dementia/Alzheimer's disease was satisfactorily valid and reliable, without systematic biases.^30^, ^31^, ^32^ Such appeared also to be the case in the present study: the CNAQ-J scores of the MD, DC, and GH groups with proxy (caregivers/family members) or surrogate (facility staff) aids were not uniformly greater/lower than those of CE group self-administered without assistance, suggesting that relevant proxy/surrogate supports may serve to enhance validity and reproducibility.

There are some limitations in the present study. As discussed above, the participants were comprised of heterogeneous Japanese elderly with respect to cognitive function, as seen in the proportions of participants with no problems (22.7%), suspected dementia (19.6%), and apparent dementia (57.7%) in the CDR analysis. These observations reflect the real-world evidence that some proportion of elderly people have a certain degree of higher brain dysfunction. However, under the present study scheme, we were unable to incisively relate cognitive levels to appetite questionnaire scores by gender, age, and instrument administration method (self-administered vs. interview-based with proxy/surrogate assistance). These issues of interest warrant further clarification using a specific research protocol.

Because we here investigated possible associations of appetite tool scores with BMIs and with nutrition-related questionnaire indices in a cross-sectional setting where causes and effects coexist at a certain point of time, we should be deliberate to draw causal inferences. Under a long-term prospective approach, with a ≥6-month observation period and sensitivity, specificity, and ROC curve analyses, we could precisely determine cutoff values, verify possible factors related to LOA, quantify its long-term effect on weight changes, and detect weight loss-associated disorders/syndromes at an early stage.

In conclusion, we developed the Japanese versions of appetite instruments: the CNAQ-J, SNAQ-J, and SNAQ-JE. Of these, the CNAQ-J and SNAQ-JE could be applied to Japanese elderly people, including those who have some cognitive impairment. Because no appreciable discrepancies were noted in reliability, validity, or reproducibility values between the two appetite tools, the SNAQ-JE appeared more feasible and practicable than the CNAQ-J for assessing appetite among the elderly, predicting declining body weight, and screening for LOA-related diseases/syndromes in the premature phase. Thus, the present data-based appetite questionnaires may contribute to elderly people's quality of life and well-being.

## Conflicts of interest

None declared.
