# Supplementary material for: Development of the Japanese version of the Council on Nutrition Appetite Questionnaire and its simplified versions, and evaluation of their reliability, validity, and reproducibility
Source: J Epidemiol. 2017 Feb 3;27(11):524–30. doi: 10.1016/j.je.2016.11.002 (PMC5608592; doi:10.1016/j.je.2016.11.002)
Supplement: Supplementary file 1 [file mmc1.pdf]

**eTable 1.** Council on Nutrition Appetite Questionnaire (CNAQ)

Administration Instructions: Ask the subject to complete the questionnaire by circling the correct answers and then tally the results based upon the following numerical scale: a=1, b=2, c=3, d=4, e=5. The sum of the scores for the individual items constitutes the CNAQ score. CNAQ score ≦28 indicates significant risk of at least 5% weight loss within six months.

1. My appetite is
   a. very poor
   b. poor
   c. average
   d. good
   e. very good
2. When I eat
   a. I feel full after eating only a few mouthfuls
   b. I feel full after eating about a third of a meal
   c. I feel full after eating over half a meal
   d. I feel full after eating most of the meal
   e. I hardly ever feel full
3. I feel hungry
   a. rarely
   b. occasionally
   c. some of the time
   d. most of the time
   e. all of the time
4. Food tastes
   a. very bad
   b. bad
   c. average
   d. good
   e. very good
5. Compared to when I was younger, food tastes
   a. much worse
   b. worse
   c. just as good
   d. better
   e. much better
6. Normally I eat
   a. less than one meal a day
   b. one meal a day
   c. two meals a day
   d. three meals a day
   e. more than three meals a day
7. I feel sick or nauseated when I eat
   a. most times
   b. often
   c. sometimes
   d. rarely
   e. never
8. Most of the time my mood is
   a. very sad
   b. sad
   c. neither sad nor happy
   d. happy
   e. very happy

eTable 2. 日本語版 CNAQ（CNAQ-J）

ここ1ヵ月間の食生活を思い出し、A〜Hの質問に対し当てはまる番号の１つに〇印をつけてください。

A. 食欲はありますか？

1. ほとんどない
2. あまりない
3. ふつう
4. ある
5. とてもある

B. 食事を、どのくらい食べると満腹感を感じますか？

1. 数口で満腹
2. 3分の１ほどで満腹
3. 半分ほどで満腹
4. ほとんど食べて満腹
5. 満腹になることはほとんどない

C. 空腹感がありますか？

1. めったに感じない
2. たまに感じる
3. 時々感じる
4. よく感じる
5. いつも感じる

D. 食事の味はいかがですか？

1. とてもまずい
2. おいしくない
3. ふつう
4. おいしい
5. とてもおいしい

E. 若いころと比べて、食事の味はどうですか？

1. とてもまずい
2. おいしくない
3. 変わらない
4. おいしい
5. とてもおいしい

F. 食事は、1日何回食べますか？

1. 1日 1回未満
2. 1日 1回
3. 1日 2回
4. 1日 3回
5. 1日 4回以上

G. 食事中に気分が悪くなったり、吐き気を感じることがありますか？

1. いつも感じる
2. よく感じる
3. 時々感じる
4. まれに感じる
5. まったく感じない

H. ふだん、どのような気持ちですか？

1. とても沈んでいる
2. 沈んでいる
3. 沈んでもなく、楽しくもない
4. 楽しい
5. とても楽しい

## eTable 3.　日本語版 SNAQ（SNAQ-J）

ここ1ヵ月間の食生活を思い出し、A～Dの質問に対し当てはまる番号の１つに○印をつけてください。

A. 食欲はありますか？

1. ほとんどない
2. あまりない
3. ふつう
4. ある
5. とてもある

B. 食事を、どのくらい食べると満腹感を感じますか？

1. 数口で満腹
2. 3分の１ほどで満腹
3. 半分ほどで満腹
4. ほとんど食べて満腹
5. 満腹になることはほとんどない

C. 食事の味はいかがですか？

1. とてもまずい
2. おいしくない
3. ふつう
4. おいしい
5. とてもおいしい

D. 食事は、1日何回食べますか？

1. 1日 1回未満
2. 1日 1回
3. 1日 2回
4. 1日 3回
5. 1日 4回以上

eTable 4. 高齢者用日本語版 SNAQ（SNAQ-JE）

ここ1ヵ月間の食生活を思い出し、A～Dの質問に対し当てはまる番号の１つに○印をつけてください。

１．食欲はありますか？

1. ほとんどない
2. あまりない
3. ふつう
4. ある
5. とてもある

2．食事を、どのくらい食べると満腹感を感じますか？

1. 数口で満腹
2. 3分の１ほどで満腹
3. 半分ほどで満腹
4. ほとんど食べて満腹
5. 満腹になることはほとんどない

３　食事の味はいかがですか？

1. とてもまずい
2. おいしくない
3. ふつう
4. おいしい
5. とてもおいしい

4．ふだん、どのような気持ちですか？

1. とても沈んでいる
2. 沈んでいる
3. 沈んでもなく、楽しくもない
4. 楽しい
5. とても楽しい
